# Absence of Neuromuscular Dysfunction in Mice with Gut Epithelium-Restricted Expression of ALS Mutation hSOD1^G93A^

**DOI:** 10.3390/biom16020253

**Published:** 2026-02-05

**Authors:** Li Dong, Xuejun Li, Ang Li, Jianxun Yi, Yanan Vockery, Yan Chang, Zui Pan, Marco Brotto, Jingsong Zhou

**Affiliations:** 1 Department of Kinesiology, College of Nursing and Health Innovation, University of Texas at Arlington, Arlington, TX 76019, USA; li.dong@uta.edu (L.D.); geraldxuejun@gmail.com (X.L.); ang.li3@uta.edu (A.L.); jianxun.yi@uta.edu (J.Y.); yanan9112@gmail.com (Y.V.); 2 Bone-Muscle Research Center, College of Nursing and Health Innovation, University of Texas at Arlington, Arlington, TX 76010, USA; yan.chang@uta.edu (Y.C.); zui.pan@uta.edu (Z.P.); marco.brotto@uta.edu (M.B.); 3 Department of Nursing, College of Nursing and Health Innovation, University of Texas at Arlington, Arlington, TX 76019, USA

**Keywords:** amyotrophic lateral sclerosis (ALS), human ALS mutation hSOD1^G93A^, skeletal muscle, transgenic mouse, gut epithelium, Cre-Lox, intestinal permeability, muscle contractility

## Abstract

Amyotrophic Lateral Sclerosis (ALS) is a devastating neuromuscular disorder characterized by the progressive loss of motor neurons and skeletal muscle, ultimately leading to respiratory failure and death, typically within 3–5 years following diagnosis. While the death of motor neurons is the pathological hallmark, ALS is increasingly recognized as a systemic disorder involving non-motor systems. Gastrointestinal dysfunction has been widely observed in both ALS patients and animal models. However, because gut abnormalities and neuromuscular degeneration are intertwined during ALS disease progression, it remains unclear whether these gut abnormalities are merely a consequence of neuromuscular degeneration or whether they play a crucial role in initiating it. In this study, we investigated whether an ALS-associated mutation expressed exclusively in the gut can directly affect neuromuscular function. We generated a novel transgenic mouse model, Gut-hG93A, which overexpresses the human ALS mutation hSOD1^G93A^ specifically in the epithelial cells of the intestine at a level comparable to the endogenous mouse SOD1. We found that the specific overexpression of hSOD1^G93A^ in gut epithelial cells did not cause abnormalities in the structure of the tight junctions or in gut permeability. Furthermore, there were no significant differences between Gut-hG93A and control mice regarding lifespan, body weight, or neuromuscular activities, including grip strength, daily travel distance and in vivo muscle contractility. These findings suggest that the ALS-associated hSOD1^G93A^ mutation, when expressed solely in the gut epithelium, is not sufficient to initiate neuromuscular degeneration of systemic ALS-like pathology.

## 1. Introduction

Amyotrophic Lateral Sclerosis (ALS) is a devastating neuromuscular disorder characterized by the progressive loss of motor neurons and skeletal muscle atrophy, leading to respiratory failure and death within 3–5 years following diagnosis [1,2]. Currently, a few FDA-approved medications targeting identified patho-mechanisms of ALS offer only limited benefits in slowing the disease progression [3,4,5]. As a complex disorder, the cause of the disease is unknown (sporadic) in most ALS patients, while familial ALS with identified mutations in various genes accounts for approximately 10% of total cases [1,5]. Both sporadic and familial ALS patients exhibit neuromuscular functional decline, suggesting common patho-mechanisms or disease modifiers. Investigating these shared mechanisms can uncover new disease modifiers, aiding in the development of ALS treatment [6].

Although the death of motor neurons is the pathological hallmark, ALS is increasingly recognized as a systemic disorder involving non-motor systems [7,8,9,10,11]. Gastrointestinal dysfunction has been observed in ALS patients [12,13,14,15,16,17,18,19], although there are some conflicting findings [20]. The advanced genetic architecture of ALS has inspired the generation of multiple animal models with ALS-associated mutations [3], significantly enhancing the understanding of the patho-mechanism underlying ALS pathogenesis and disease progression. Particularly, the ALS mouse model with overexpression of human ALS mutation hSOD1^G93A^ (G93A) ubiquitously in all tissues effectively recapitulates many features of the human disease and is the most characterized and extensively used in ALS basic and preclinical studies [21,22]. An earlier investigation using the G93A mouse model revealed a leaky intestine with disrupted tight junctions. Notably, this type of gut dysfunction occurred early, before the onset of ALS neuromuscular symptoms [8]. A later longitudinal study of G93A mice also reported that abnormalities of microbiota were evident by 37 days of age, which preceded the onset of neuromuscular function decline [23]. Reducing the gut microbial burden in the mutant C9orf72 ALS mouse model through broad-spectrum antibiotic treatment attenuated inflammatory phenotypes [24]. Interestingly, the G93A mouse model under broad-spectrum antibiotic treatment or germ-free conditions showed exacerbated disease progression with worsened motor function and decreased survival [25,26]. Despite some controversial observations, the close interrelation between gut abnormalities and ALS pathogenesis has gained significant interests, as highlighted in recent review articles [27,28,29,30,31,32,33].

While gut abnormalities and neuromuscular degeneration are intertwined during ALS disease progression, it remains unclear whether these gut abnormalities are merely a consequence of the neuromuscular degeneration or if they play a crucial role in initiating it. To directly test these hypotheses, we investigated whether an ALS mutation expressed solely in the gut can directly affect neuromuscular function. We generated a new transgenic mouse model (Gut-hG93A) with overexpression of the human ALS mutation hSOD1^G93A^ exclusively in the epithelial cells of the intestine. We examined whether the ALS mutation hSOD1^G93A^ could directly impact gut epithelial function and subsequently cause a decline in neuromuscular function.

## 2. Materials and Methods

### 2.1. Transgenic Mouse Models and Genotyping

The ALS transgenic mouse model (hSOD1^G93A^, or G93A for short) with genetic background of B6SJL was originally generated by Drs. Deng and Siddique’s group at Northwestern University and deposited to the Jackson Lab as B6SJL-SOD1^G93A^ [21]. The wild-type (WT) B6SJL, B6SJL-SOD1^G93A^ and Vil1-Cre mice were purchased from Jackson Laboratory (#100012, #002726 and #021504). We maintained G93A mouse line through breeding B6SJL/G93A male mice with B6SJL/WT female mice.

The transgenic Lox-SOD1^G93A^ mouse line was generated through working with Cyagen, California. The cDNA of human SOD1^G93A^ was a kind gift from Dr. Hang-Xiang Deng at Northwestern University, Chicago. This hSOD1^G93A^ was used to construct the plasmid pRP-LoxStopLox-hSOD1^G93A^, which was used to generate the transgenic Lox-SOD1^G93A^ (Lox-hG93A) founder mice by Cyagen. To maintain the consistency of the genetic background, the mice of backgrounds other than B6SJL, such as Vil1-Cre, were backcrossed with B6SJL for at least 5 generations to change their genetic background to B6SJL.

All mice other than WT were genotyped at weaning by extracting genomic DNA from the tail tip and carrying out PCRs with these primers. An internal control primer set targeting the mouse interleukin-2 (IL-2) gene was included in all genotyping reactions to confirm DNA integrity and PCR efficiency. The primers used were as follows: (1) for SOD1^G93A^ mouse, forward 5′CATCAGCCCTAATCCATCTGA3′, reverse 5′CGCGACTAACAATCAAAGTG3′; (2) for Vil1-Cre mouse, forward 5′GCCTTCTCCTCTAGGCTCGT3′, reverse 5′AGGCAAATTTTGGTGTACGG3′; and (3) for Lox-SOD1^G93A^ mouse, forward 5′CATCAATTTCGAGCAGAAGGAAAG3′, reverse 5′TGCTTTTTCATGGACCACCAG3′.

### 2.2. Immunohistochemistry

The immunohistochemistry protocol was adapted from our previous publication [34]. The intestinal tissue was dissected from the experimental mice and fixed in 4% paraformaldehyde (PFA) overnight at 4 °C. After washing with PBS, tissues were dehydrated through a graded series of alcohol. Followed by xylene, paraffin embedding was carried out under negative pressure for 3 h. Samples were cut into 8 µm sections for immunostaining. Antigen retrieval was carried out at 95 °C for 20 min in citrate buffer (pH 6.0). Samples were blocked at room temperature for 2 h with blocking solution containing 3% goat serum, 3% BSA, 0.1% Tween-20, 0.1% Triton X-100 and 0.1% NaN3. The samples were then incubated with anti-E-cadherin (1:200) (Cell Signaling Technology, Danvers, MA, USA; Cat# 14472S) or anti-SOD1 (1:200) (Proteintech, Rosemont, IL, USA; Cat# 10269-1-AP) at 4 °C overnight. After washing with PBS, the samples were incubated with Alexa Fluor conjugated secondary antibodies of the corresponding species (1:1000). After washing with PBS, samples were mounted in anti-fade mounting medium containing 60% glycerol and 0.5% N–propyl gallate for imaging under Leica TCS SP8 confocal microscope (Leica Microsystems Inc., Wetzlar, Germany).

### 2.3. Immunoblotting Assay

Western blot was performed as previously described [35]. Protein samples extracted from mouse tissue were separated via SDS-polyacrylamide gel electrophoresis (SDS-PAGE), followed by transferring to a PVDF membrane with a Bio-Rad semidry transfer cell (1703940, Plano, TX, USA). After blocking with 5% milk in Tris-buffered saline plus 0.1% Tween-20 (TBST) at room temperature for 1 h, membranes were incubated with primary antibodies at 4 °C. The primary antibodies used in this study were anti-SOD1 (1:3000) (Proteintech, Rosemont, IL, USA. Cat#10269-1-AP), anti-E-cadherin (1:3000) (Cell Signaling Technology, Danvers, MA, USA. Cat#14472S), ZO-1 (1:3000) (Invitrogen, Carlsbad, CA, USA. Cat# 402300), and anti-GAPDH (1:5000) (Proteintech, Rosemont, IL, USA. Cat# 10494-1-AP). After washing with TBST buffer, the membranes were incubated with horseradish-peroxidase-conjugated secondary antibody at room temperature for 2 h. Protein bands were visualized using a Bio-Rad Clarity ECL kit under the ChemiDoc Imaging System (Bio-Rad Laboratory, Hercules, CA, USA). The band intensity was analyzed using ImageJ software (version 1.54p).

### 2.4. Gut Permeability Assessment

The protocol for measuring gut permeability was adapted from previous publications [8,36]. The fluorescent-labeled dextran dyes, fluorescein isothiocyanate (FITC)-4KD (FD4) with MW 4000 (Sigma, St. Louis, MO, USA; Cat# FD4) and rhodamine B isothiocyanate (R70) with MW of 70,000 (Sigma, St. Loius, MO, USA; Cat# R9379), were dissolved in PBS solution at concentrations of 40 mg/mL and 16 mg/mL, respectively. A dose of 27 mg FD4 and 16 mg R70 per 100 g mouse body weight was used for oral gavage. Briefly, the experimental mouse was weighed first. After 4 h of fasting, the first blood sample was collected via tail vein. Then, a mixture of FITC (FD4) and rhodamine B isothiocyanate (R70) solution was gently gavaged using a 20 G feeding tube (Pet Surgical, Sherman Oaks, CA, USA). The mouse was returned to its cage with water supply for another 3 h, then the second blood sample was collected. The serum samples were used to determine the fluorescence intensity of FITC (FD4) (Ex485/Em528) and rhodamine B isothiocyanate (R70) (Ex530/Em645) using a SpectraMax i3x (Molecular Devices, San Jose, CA, USA).

### 2.5. In Vivo Muscle Contractility Assessment

In vivo muscle contractility assessments were performed using the whole Muscle Test System, Model 1300A (Aurora Scientific, Inc., Aurora, ON, Canada), following the manufacturer’s instructions. The experimental mouse was initially anesthetized via isoflurane inhalation (4% for induction and 2–2.5% for maintenance of anesthesia) and placed on a heated platform to maintain a stable body temperature. The hair on the hind limb was removed, and the area was cleaned with 70% alcohol. The limb was stabilized on the testing rig using a knee clamp. The mouse’s foot was secured to the footplate with medical tape. The footplate, which was attached to a torque sensor, was initially aligned orthogonally (at 90°) to the tibia. One subcutaneous stimulating electrode was placed near the neck of the fibula to target the fibular nerve. Another electrode was placed near the gastrocnemius muscle on the side of the leg. The leg was carefully positioned to prevent simultaneous stimulation of opposing muscles and to ensure the maximum force could be achieved upon stimulation. The optimal stimulation frequency was determined by progressively increasing the pulse frequency from 20 to 200 Hz over a 200 ms interval. A maximal fused tetanic contraction was typically achieved between 125 and 150 Hz. Following the experiment, the electrodes were removed, and the knee clamp was unlocked. The mouse was returned to its home cage upon recovery of normal ambulation.

### 2.6. Mouse Daily Activity Monitoring

Daily locomotor activity of the experimental mice was evaluated using the PhenoSys Activity Monitor (Berlin, Germany), following the manufacturer’s instructions. Each mouse was first anesthetized via isoflurane inhalation (4% for induction and 2–2.5% for maintenance of anesthesia). A radio-frequency-identification (RFID) transponder (planet-ID GmbH, Essen, Germany) was then implanted into the back of the mouse. The home cage containing the experimental mice was placed on top of an RFID sensor plate. This setup allows the mice to move freely within the cage, enabling continuous monitoring of their activity throughout the day. For reliable individual activity recording, up to four mice were housed in a single cage. To minimize environmental and social disturbance, it was essential that the experimental mice had been previously socialized with each other, being housed together for the recording period. The distance traveled during the specified period was calculated using the proprietary Analytics software (version 210421) provided by the manufacturer.

### 2.7. Grip Strength Assessment

Grip strength was measured using the GS3 meter (Bioseb, Vitrolles, France), following the manufacturer’s instructions. The test determined the maximal muscle force exerted by all four limbs (combined force of both forelimbs and hind limbs). The mouse was held by the tail and placed on a metal grid. Once the mouse securely grasped the metal grid with all four limbs, it was gently pulled along the axle of the sensor until it released the grid. Five consecutive tests were performed for each mouse. The average force value from these trials was used for final analysis.

### 2.8. Statistical Analysis

Data are presented as mean ± S.E.M (standard error of the mean). Statistical comparisons between two groups were performed using a two-sided Students’ *t*-test. A *p*-value of *p* < 0.05 was considered statistically significant. Line plots were generated using Excel. Box-and-whisker plots were generated in RStudio (version 2025.09.2) using ggplot2 package. The box bottom, median line, and box top represent the 25th (Q1), 50th (Q2), and 75th percentile (Q3), respectively. The whiskers extend to Q1 − 1.5 × IQR and Q3 + 1.5 × IQR, respectively. IQR is interquartile range (Q3–Q1). Survival curves were analyzed and generated using GraphPad Prism 10.0 software.

## 3. Results

### 3.1. Generation of Transgenic Mice with Gut-Specific Overexpression of ALS Mutation hSOD1^G93A^ (Gut-hG93A)

**Figure 1:**
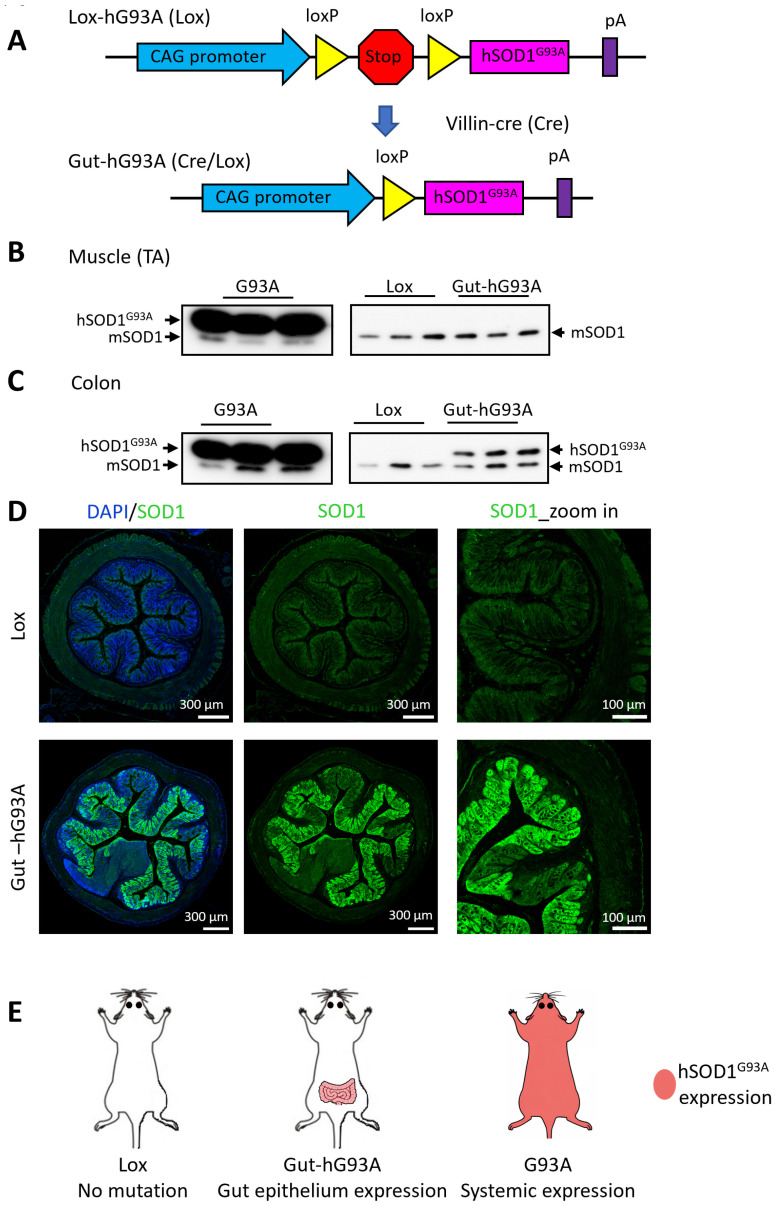
Development of Gut-hG93A mouse model with gut-specific hSOD1G93A expression. (**A**) Schematic detailing generation of Lox-hG93A (Lox) mice and subsequent cross-breeding with Villin–Cre (Cre) mice to produce Gut-hG93A (Cre/Lox) mice, with hSOD1^G93A^ overexpression restricted to intestinal epithelial cells under Villin 1 promotor. (**B**) Immunoblotting of tibialis anterior muscle (TA) using pan-anti-SOD1 antibody (recognizing both human and mouse SOD1) confirms the hSOD1^G93A^ expression only in G93A mice, not in Lox and Gut-hG93A mice. (**C**) Immunoblotting of colon samples shows hSOD1^G93A^ expression in Gut-hG93A mice, at a level comparable to native mouse mSOD1 (*n* = 3, unpaired *t*-Test, *p* > 0.05). (**D**) Representative IHC images of using the anti-SOD1 antibody (green) reveal basal native mouse mSOD1 expression in the colon of Lox mice, and enhanced mosaic expression of hSOD1^G93A^ in the colon of Gut-hG93A mice. Nuclei stained with DAPI (blue). (**E**) Illustration of the different expression of hSOD1^G93A^ between the Lox, Gut-hG93A, and systemic G93A mice. Figure S1 has Original images for Figure 1B–D.

To achieve tissue-specific overexpression of the human ALS mutation hSOD1^G93A^ exclusively in the gut, we employed a Cre-Lox system. A plasmid (pRP-LoxStopLox-hSOD1^G93A^) was engineered containing a STOP cassette flanked by two LoxP sites. This cassette was inserted between the hybrid CAG promoter and the hSOD1^G93A^ transgene (Figure 1A). This plasmid was used to generate transgenic founder mice, designated as Lox-hG93A (Lox). In these mice, the LoxP-STOP-LoxP sequence prevents the expression of the hSOD1^G93A^ transgene. We purchased Villin–Cre mice (Cre) from Jackson Laboratory (#021504). In these mice, the mouse Villin 1 promoter directs the expression of Cre recombinase specifically in the villus of crypt epithelial cells in the small and large intestines, closely mimicking the native Vil1 expression level (https://www.jax.org/strain/021504, accessed on 15 December 2025). By crossing the Lox-hG93A (Lox) mice with the Villin–Cre mice, we generated double transgenic mice, referred to as Cre/Lox-hG93A (Gut-hG93A). In the presence of Cre recombinase, the LoxP sites facilitate recombination, leading to the excision of the stop codon cassette [21,37]. This excision results in the Cre-dependent and exclusive expression of the hSOD1^G93A^ transgene within the epithelial cells of the intestine in the Gut-hG93A mice.

### 3.2. Confirmation of Gut-Specific hSOD1^G93A^ Expression

The gut-specific overexpression of hSOD1^G93A^ in the Gut-hG93A mice was confirmed through immunoblotting and immunohistochemical (IHC) assessments using an antibody recognizing both human and mouse SOD1. As illustrated in Figure 1B,C, the native mouse SOD1 protein (mSOD1) was detected by the antibody in both skeletal muscle (Tibialis Anterior) and colon samples from all tested mouse lines (ALS G93A, Lox-hG93A (Lox), and Gut-G93A). The forced, intensive expression of the human SOD1^G93A^ protein (hSOD1^G93A^) was detected in both skeletal muscle and colon samples of the standard G93A mice, which ubiquitously overexpress hSOD1^G93A^ in all tissues [21,37] (Figure 1B,C, left panels). The Lox-hG93A mice showed an absence of hSOD1^G93A^ in both skeletal and colon tissues (Figure 1B,C, right panels). This confirmed the successful blockage of hSOD1^G93A^ expression by the LoxP-Stop-LoxP sequence in the Lox-hG93A mice. Consequently, the Lox-hG93A (Lox) mice were used as the negative control for all remaining experiments in this study. Critically, the Gut-hG93A mice exhibited hSOD1^G93A^ expression in the colon but not in the skeletal muscle (Figure 1B,C, right panels), confirming the successful, specific overexpression of transgene hSOD1^G93A^ in the intestine. The expression level of hSOD1^G93A^ in the colon of Gut-hG93A mice was found to be similar to that of native mSOD1 (Figure 1C, right panel). The ratio of hSOD1^G93A^ band intensity to mSOD1 was approximately 1.5; although this difference was not statistically significant (*p* = 0.1, unpaired *t*-Test, *n* = 3). This result is consistent with expectation, as the Villin 1 promoter is known to induce the exogenous gene expression in a pattern closely resembling native expression (The Jackson Laboratory, Bar Harbor, ME, USA; Strain 021504).

To further characterize the tissue-specific expression, immunohistochemical (IHC) analysis was performed on colon samples from both Lox-hG93A (Lox) and Gut-hG93A mice (Figure 1D). Identical preparation steps, including antibody concentration and incubation conditions, were applied to samples of both groups. Under the same confocal microscope setting, the colon sections of control Lox mice showed homogeneous expression of the native mSOD1 within the villi (green fluorescence). In contrast, colon sections from the Gut-hG93A mice exhibited a marked increase in SOD1 green fluorescence within the villi, confirming the successful expression of exogenous hSOD1^G93A^ transgene. The expression of hSOD1^G93A^ was not homogeneous but displayed some mosaic or patchy patterns, which is an observation consistent with previous reports using Villin 1 transgenic mice [38]. Thus, both the immunoblotting and IHC results confirm the specific and conditional overexpression of hSOD1^G93A^ restricted to the intestinal epithelia of Gut-hG93A mice.

### 3.3. Assessment of Gut Barrier Function in Gut-hG93A Mice

Previous studies have reported that standard ALS G93A mice exhibit compromised gut barrier function, characterized by increased gut permeability and disrupted epithelial tight junction, compared to age-matched wild-type mice [8]. Here, we investigated whether the specific overexpression of the ALS mutation hSOD1^G93A^ restricted to the gut epithelial cells in the Gut-G93A mice would be sufficient to cause the similar gut permeability phenotypes observed in the ubiquitous G93A model.

Oral gavage with fluorescent-conjugated dyes, followed by measuring their presence in the circulating blood, is a common and quantitative method to evaluate intestinal permeability in animal models [36]. Intestinal permeability is generally categorized based on the size of the molecules passing across the epithelial barrier. There are two paracellular permeability pathways: the pore pathway restricted to small molecules (<6 Å) and the leak pathway allowing passage of larger molecules (<100 Å), both mediated by tight junctions. This pathway was assayed using the fluorescent labeled dextran dye, fluorescein isothiocyanate (FITC)-4KD (FD4), which has a size of approximately 28 Å. The unrestricted pathway is characterized by passage of larger molecules (>100 Å), typically caused by epithelial damage [39]. This pathway was assayed using the fluorescent dye rhodamine B isothiocyanate (R70) [36].

**Figure 2:**
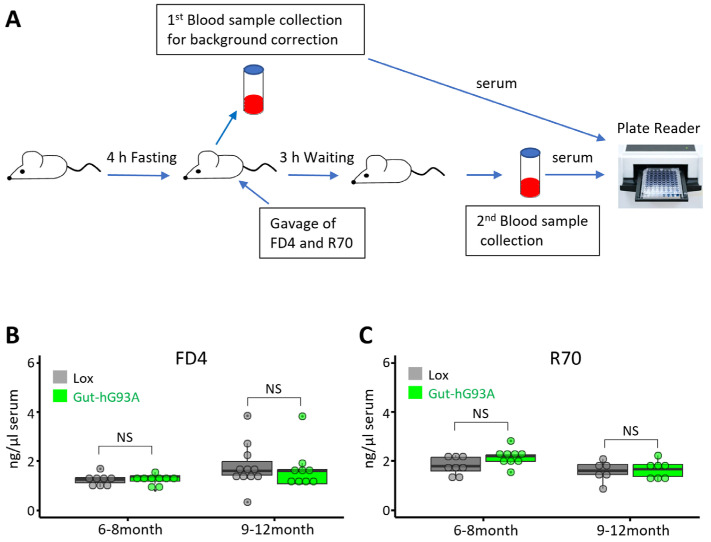
Gut-hG93A mice exhibit no detectable changes in gut permeability. (**A**) Schematic of the gut permeability assay. Mice (6–8 and 9–12 months) were fasted for 4 h, followed by the first baseline blood sample collection (50 µL), then given oral fluorescent tracers (FD4 and R70). After 3 h, a second blood sample was collected, and serum fluorescence was measured by Plate Reader. (**B**) Serum FD4 (4 kDa) levels did not differ between Lox and Gut-hG93A mice (*n* = 8–11, unpaired *t*-test, *p* > 0.05). (**C**) Serum R70 (70 kDa) levels also show no difference between groups (*n* = 8–11, unpaired *t*-test, *p* > 0.05). NS stands for non-significant.

To evaluate both the leak and unrestricted pathways simultaneously, FD4 and R70 were combined in the same gavage application. Because FD4 and R70 have distinct excitation and emission wavelengths, their fluorescence signals could be detected simultaneously in the serum samples using a Spectrophotometer Plate Reader (Molecular Devices, San Jose, CA, USA). As illustrated in Figure 2A, the experimental mice were fasted for 4 h prior to the procedure to avoid potential interference from feeding. Blood samples (50 µL each) were collected immediately before gavage with the dye solution. The dye solution was administered via oral gavage. A second set of blood samples was collected three hours later. The fluorescence of FD4 and R70 in the serum samples was recorded. The readings of serum samples without gavage of fluorescent dyes were used to correct the autofluorescence background. The serum levels of FD4 and R70 in the Gut-hG93A mice were compared to the Lox-G93A (Lox) control mice. As shown in Figure 2B, the systemic leakage of FD4 and R70 into the bloodstream was not significantly different between the Lox and Gut-hG93A mice at two different age points: 6–8 months old (*n* = 8 Lox, 9 Gut-hG93A, *p* = 0.78) and 9–12 months old (*n* = 11 Lox, 9 Gut-hG93A, *p* = 0.76).

These results indicate that the gut-specific overexpression of hSOD1G93A in the Gut-hG93A mice does not cause a detectable change in intestinal permeability via the leak or unrestricted pathways at these ages.

### 3.4. Analysis of Epithelial Tight Junctions and Adherens Junctions

The gut barrier function is critically dependent on the integrity of apical junction complex, which involves multiple proteins such as E-cadherin (a main component of adherens junction) and ZO-1 (a main component of tight junction) [39]. We investigated whether the gut-specific overexpression of hSOD1^G93A^ altered the expression of these proteins in the colon.

**Figure 3:**
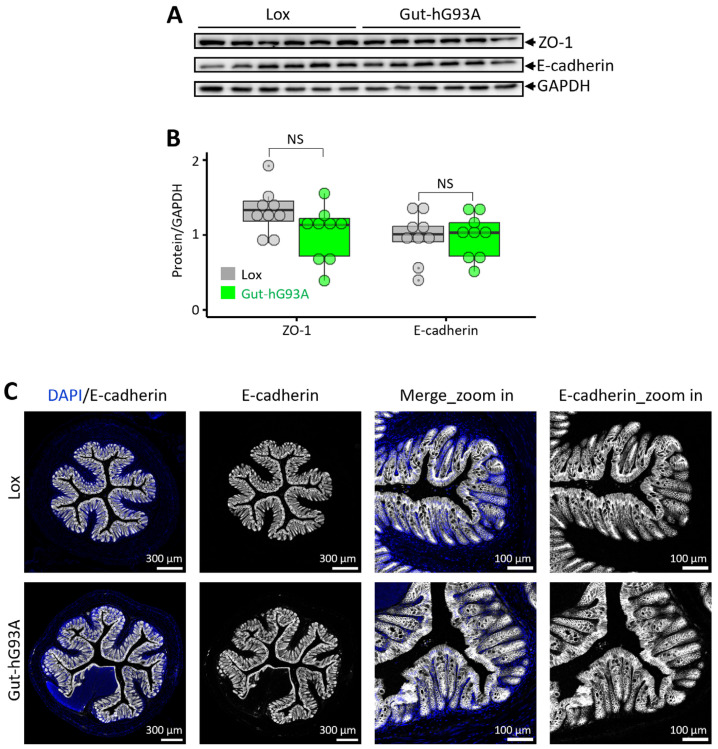
Gut-hG93A mice exhibit no detectable changes in expression levels of tight junction proteins and the villi morphology. (**A**) The immunoblotting analysis of E-cadherin and ZO-1 in colon samples from Lox and Gut-hG93A mice. (**B**) Quantitation shows no significant difference (*n* = 9, unpaired *t*-test, *p* > 0.05). (**C**) Representative IHC images of E-cadherin (gray) reveal similar colon (left two panels) and villi (two zoom-in panel on right) morphology between groups. DAPI (blue) stains the nuclei. NS stands for non-significant Figure S2 has Original images for Figure 3A,C.

The immunoblotting was performed on colon tissue to compare the expression level of E-cadherin and ZO-1 proteins between Gut-hG93A and the Lox control mice (Figure 3A). As shown in Figure 3B, no significant difference in protein expression was identified between the two genotypes at the age of 18 months (*n* = 9, *p* = 0.91 for E-cadherin, and *p* = 0.08 for ZO-1).

Further immunohistochemical studies were performed using an anti-E-cadherin antibody to investigate the expression pattern of E-cadherin within the colon epithelial layer. DAPI was used to stain and visualize nuclei. The representative images are shown in Figure 3C. The expression level and pattern of E-cadherin were found to be similar in the colon of both Gut-hG93A and Lox mice.

These results, derived from both immunoblotting and IHC, collectively confirm the absence of detectable morphological or expression changes in the key epithelial junction proteins caused by ALS hSOD1^G93A^ mutation when expressed solely in the gut epithelial cells.

### 3.5. Neuromuscular Activity Assessment in Gut-hG93A Mice

Although our initial experiments showed no detectable changes in gut permeability or tight junction protein expression in the Gut-hG93A mice, we could not exclude the possibility of other functional changes in the gut that may subsequently impact systemic neuromuscular function. To thoroughly assess the overall neuromuscular activity of the experimental mice, we employed a comprehensive matrix of in vivo assessments, including grip strength, in vivo contractility and daily movement tracking. Given that gender plays a significant role in neuromuscular function, particularly during the disease progression of ALS G93A mice [40,41], the neuromuscular activities of experimental mice were assessed and analyzed separately based on gender.

**Figure 4:**
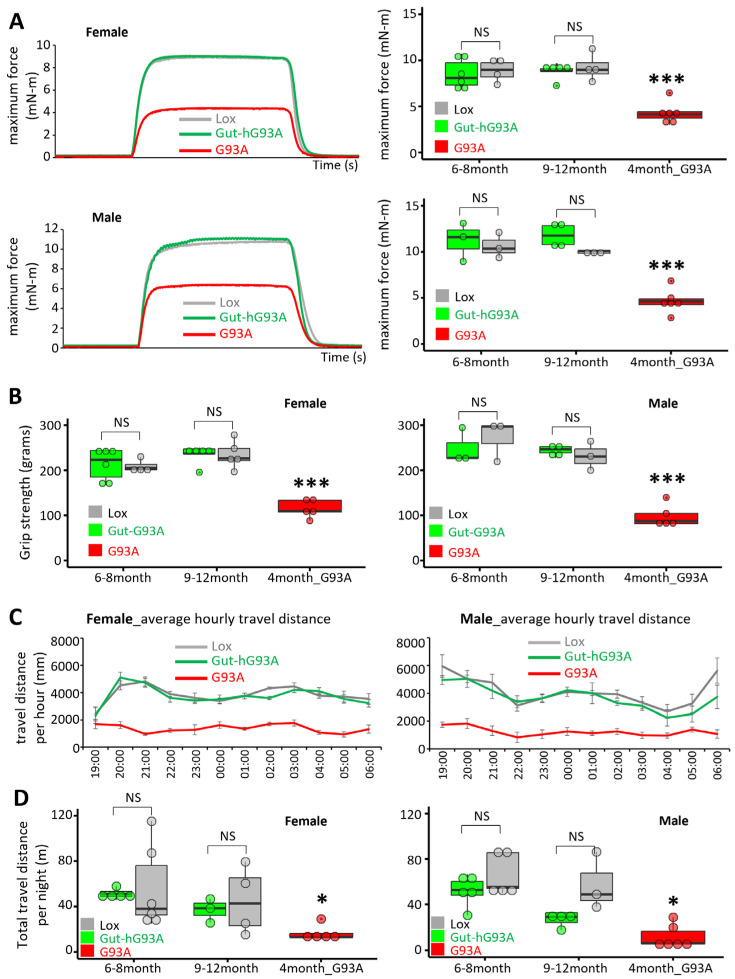
Gut-hG93A mice exhibit no abnormalities in neuromuscular activities. (**A**) Maximum force (mN-m) recorded in female (top) and male (bottom) mice from Gut-hG93A, Lox, and G93A groups using in vivo contractility measurement. Gut-hG93A and Lox mice were tested at 6–8 and 9–12 months; G93A mice at 4 months served as positive control (left panels). Quantification (right panels) shows G93A mice had significantly reduced maximum force compared to Lox and Gut-hG93A (*n* = 4–6, one-way ANOVA with Tukey post hoc; *p* < 0.005), with no difference between Gut-hG93A and Lox (*p* > 0.05). (**B**) Grip strength was measured in female and male mice. G93A mice showed a significant reduction, while Gut-hG93A and Lox did not differ (*p* > 0.05). (**C**) Hourly travel distance recorded from 7 p.m. to 6 a.m. averaged over 4 weeks. Data were collected from Gut-hG93A and Lox mice with the ages ranging from 6 to 12 months (*n* = 8 male and 8 female Gut-hG93A, *n* = 11 female and 5 male Lox), and 3- to 4-month-old G93A mice (*n* = 5–6). (**D**) Total travel distance. G93A mice showed reduced total travel distance (*n* = 5–6, *p* < 0.05), while Gut-hG93A and Lox did not differ (*n* = 4–6, *p* > 0.05). * *p* < 0.05, *** *p* < 0.001. NS stands for non-significant.

The Aurora Whole Muscle Test System was used to measure the in vivo contractility of the plantar flexion muscle in live mice. The representative maximum forces for female and male groups are shown in Figure 4A (left panels). No significant difference in maximum force was observed between the Gut-hG93A mice and the Lox control mice in either gender at two tested age windows (6–8 months and 9–12 months) (Figure 4A, right panels). Furthermore, analysis of variance (ANOVA) revealed no significant difference between these 6–8-month and 9–12-month age groups. Specifically, the *p*-values for the main effect of genotype were *p* = 0.41 for males and *p* = 0.86 for females. The maximum forces measured in G93A mice at the age of 4 months were included as positive control. These data clearly reflect the detectable defects in neuromuscular function characteristic of the ALS G93A model, providing a benchmark against which the negligible effects in Gut-G93A mice could be compared.

The grip strength, which measures the maximal combined force of all four limbs, was compared between Gut-hG93A mice and the Lox control mice. Assessment was conducted separately for male and female mice at two age windows: 6–8 months and 9–12 months. As shown in Figure 4B, no significant difference in grip strength was observed between the Gut-hG93A and the Lox control mice in either male or female cohorts at both age windows. Analysis of variance (ANOVA) further indicated no significant difference in grip strength between the two age groups for the Lox and Gut-hG93A mice combined. ANOVA *p*-values for main effect of genotype (Lox vs. Gut-hG93A): males *p* = 0.52 and females *p* = 0.40. The grip strength of G93A mice at the age of 4-months-old was used as the positive control for reduced grip strength. Both male and female G93A mice showed a significant reduction in grip strength when compared to both the Lox and Gut-hG93A mice (ANOVA *p*-values for comparison against G93A: males *p* = 9.3 × 10^−6^, females *p* = 2.9 × 10^−6^).

Using the PhenoSys Activity Monitor (PhenoSys GmbH, Berlin, Germany), the movement of individual mice in their home cage was tracked continuously for 24 h. We compared the daily movement of Gut-hG93A and Lox control mice. Given the absence of significant difference between the two age groups (6–8 months and 9–12 months) in the in vivo contractility and grip strength assays, the daily movements data were pooled across these age groups for analysis. Figure 4C shows the time course of the average hourly travel distance (calculated over 4 weeks) during nocturnal hours for both male and female cohorts. While locomotor activity was recorded continuously over a 24 h cycle, our primary travel distance analysis focused on the night hours (7:00 p.m.–7:00 a.m.). This approach was taken to prioritize the mice’s period of peak activity during the dark phase and to eliminate potential interference from human presence in the animal facility during standard working hours. Importantly, we confirm that daytime (diurnal) activity levels were comparable between Gut-hG93A and Lox control mice, with both groups exhibiting the expected minimal activity typical of the resting phase.

The average hourly travel distance of ALS G93A mice was used as a positive control. We used data from G93A mice recorded between 3 and 4 months of age, as this stage is known to precede paralysis and demonstrate a significantly reduced daily movement compared to Lox or Gut-hG93A mice in both genders. Importantly, there were no significant differences in the average hourly travel distance between the Gut-hG93A and the Lox control mice in either gender. We also compared the total travel distance per day during the dark cycle (Figure 4D). While the total travel distance was significantly reduced in the G93A mice, no difference was observed between Lox and Gut-hG93A mice.

These comprehensive data confirm that the gut-specific overexpression of hSOD1^G93A^ has no detectable effect on the overall locomotor activity or neuromuscular performance of the animals.

### 3.6. Impact on Survival and Body Weight of Gut-hG93A

**Figure 5:**
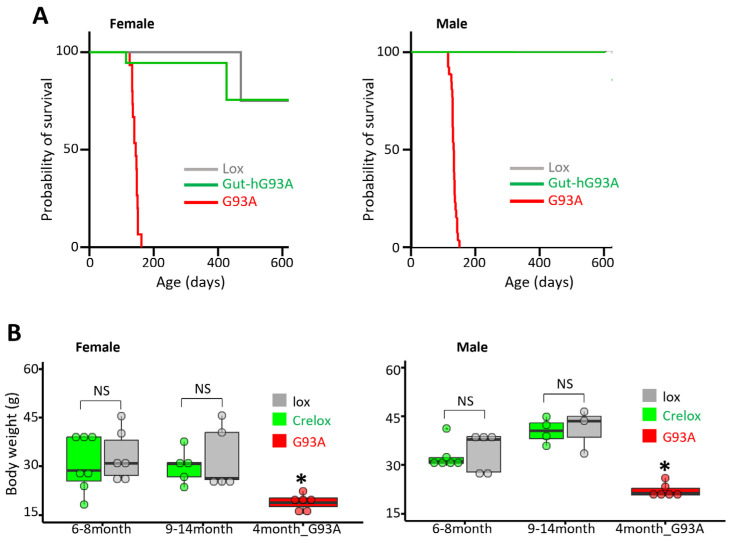
Gut-hG93A mice show no significant reductions in lifespan and body weight compared to the control Lox mice. (**A**) The lifespan of both male and female Gut-hG93A mice is not different from the control Lox mice (male: *n* = 11 Gut-hG93A, 13 Lox; Female: *n* = 18 Gut-hG93A, 15 Lox; log-rank test, *p* > 0.05), while G93A mice has much shorter lifespan (<200 days) (*n* = 26 male, 15 female; *p* < 0.0001). The lifespan curves of male Lox and Gut-hG93A are overlapped. All deaths observed during the study were natural. Mice that were alive at the predefined experimental endpoint of 600 days were administratively censored. The endpoint (death) of G93A mice was defined by the loss of righting reflex within 30 s when the mouse was placed on its side. (**B**) Body weight analysis reveals no difference between gut-hG93A and Lox mice in both female (*n* = 12 Gut-hG93A and 11 Lox; *p* > 0.05) and male (*n* = 12 Gut-hG93A and 13 for lox; *p* > 0.05) mice. In contrast, both male and female G93A mice (*n* = 6/group) show significant reductions in body weight (one-way ANOVA with Tukey post hoc; *p* < 0.001). * *p* < 0.05. NS stands for non-significant.

We evaluate whether the gut overexpression of the human ALS mutation hSOD1^G93A^ in Gut-hG93A mice impacts overall body metabolism and lifespan. We compared a cohort of 31 Gut-hG93A mice (19 females and 12 males) with 28 Lox control mice (15 females and 13 males). The majority of both control and Gut-hG93A mice survived beyond 600 days (Gut-hG93A: 16/19 females and 10/12 males; Lox: 13/15 females and 12/13 males). As shown in the survival curves (Figure 5A), a log-rank test (*p* = 0.7943) showed no significant difference in lifespan between Gut-hG93A and Lox mice when analyzed by gender. The lifespan data for 15 female and 26 male G93A mice were included as a positive control, demonstrating a significantly shorter lifespan, with all G93A mice having a lifespan shorter than 200 days.

Body weight was compared between Gut-hG93A and Lox mice at two age windows, with G93A mice (4-month-old) used as a positive control. No significant difference in average body weight was observed between Gut-hG93A and Lox mice in either gender. In contrast, the average body weight of the G93A mice at the age of 4 months was significantly reduced (Figure 5B).

Our data collectively indicate that the gut-specific expression of the ALS mutation hSOD1^G93A^ has no substantial negative impact on whole-body metabolism or the lifespan of the experimental mice up to the age of 600 days.

## 4. Discussion

In the current study, we successfully generated and characterized a novel transgenic mouse model, Gut-hG93A, which overexpresses the ALS-associated mutation hSOD1^G93A^ exclusively in the intestinal epithelial cells of villi. Comprehensive analysis revealed that the gut epithelium-restricted expression did not lead to detectable abnormalities in tight junction structure or overall intestinal permeability. Furthermore, compared to the Lox-hG93A (Lox) control mice, Gut-hG93A mice showed no significant differences in lifespan, body weight, grip strength, daily travel distance, or in vivo muscle contractility.

Immunoblotting, using an antibody recognizing both mouse and human SOD1, confirmed the restricted overexpression of hSOD1^G93A^ in the gut of Gut-hG93A mice, at a level comparable to the endogenous mouse SOD1 (Figure 1B,C). While immunohistochemistry revealed a mosaic expression pattern within the intestinal villi (Figure 1D), a documented characteristic of the Villin 1 promoter [38], our immunoblotting data provide quantitative confirmation that the total protein burden reaches ~1.5:1 ratio with the native protein. It is theoretically possible that regions with lower expression could partially mitigate localized cellular stress. However, given that the intestinal barrier functions as a coordinated syncytium, a ~1.5:1 ratio of mutant protein across the bulk tissue would typically be expected to induce measurable shifts in tight junction integrity or cytokine signaling if the mutation possessed a strong epithelial-autonomous toxicity. The total absence of functional deficits (Figure 2), despite the widespread presence of mutant protein, suggests that this expression pattern does not mask a latent pathogenic effect. Instead, it suggests that hSOD1^G93A^ is not a primary driver of barrier failure at these physiological levels.

Importantly, no structural disruption of colonic villi was observed. Furthermore, expression levels of the tight junction proteins ZO-1 and E-cadherin, as well as E-cadherin spatial distribution, remained unchanged (Figure 3). Consistent with these findings, gut permeability was not increased in Gut-hG93A mice (Figure 2), standing in stark contrast to the systemic ALS G93A mouse model, which exhibits early-stage gut barrier dysfunction [8]. We conducted a series of rigorous neuromuscular behavior tests and lifespan evaluations to assess any potential systemic dysfunction originating from the gut-restricted expression of hSOD1^G93A^. Up to 600 days of age, Gut-hG93A mice showed no difference in survival compared to the Lox control mice. This contrasts sharply with the standard G93A mice, which displayed markedly shortened lifespan and severe weight loss by 4 months (Figure 5). In vivo muscle contractility tests, which measure maximal force in response to electrical stimulation under anesthesia (reflecting a pure muscle response), showed no difference in the maximal force between Gut-hG93A and Lox control mice up to 12 months of age, indicating muscle function remained unaltered in Gut-hG93A mice (Figure 4A,B). Similarly, grip strength and daily travel distance remained comparable (Figure 4C,D). Collectively, these data demonstrated that gut villi-overexpression of the ALS-associated mutation hSOD1^G93A^ does not impair intestinal barrier integrity, neuromuscular function, or influence lifespan in Gut-hG93A mice.

A critical consideration in interpreting our findings is the relatively low mutant protein burden in the Gut-hG93A model compared to the standard systemic G93A ALS model. The G93A mouse model carries high copy numbers of the hSOD1^G93A^ transgene systemically across all tissues, resulting in mutant protein expression up to 24-fold higher than endogenous mouse SOD1 [21,42] (Figure 1B,C). Given that ALS disease severity is strictly dose dependent with low-copy models exhibiting delayed disease onset and progression [42,43,44], the physiological expression levels in our Gut-hG93A mice (roughly 1.5:1 ratio with endogenous SOD1) may fall below the pathological threshold required to trigger protein aggregation or systemic decline within the observed timeframe. While this lower expression burden more accurately reflects mutant protein levels in human ALS patients, it likely limits the model’s ability to drive rapid pathology. It remains to be determined whether dose-escalation—through the generation of higher-copy number lines or localized viral overexpression—would reveal a critical threshold where epithelial hSOD1^G93A^ becomes sufficient to initiate barrier failure. Moreover, the lack of a detectable phenotype suggests that at physiological levels, gut epithelium-restricted expression of hSOD1^G93A^ alone is insufficient to act as a primary driver of the disease.

It remains possible that pathological changes in Gut-hG93A mice could emerge beyond 600 days. Considering that one human year approximates nine mouse days [45], a 600-day-old mouse corresponds to a 66-year-old human. ALS can occur at any age, but it typically manifests between 45 and 75 years [46], with familial ALS onset averaging 40–60 years and sporadic ALS 58–63 years [47]. Under our experimental condition, Gut-hG93A mice lived well into the high-risk range for ALS onset. Thus, we conclude that the physiological level expression of ALS-associated hSOD1^G93A^ in gut villi does not cause detectable damage to intestinal structure and function, nor does it induce neuromuscular dysfunction.

Because hSOD1^G93A^ expression in gut villi of Gut-hG93A mice is comparable to native SOD1, one might speculate that native wild-type mouse SOD1 compensates for mutant toxicity. However, the existing literature suggests otherwise. SOD1 is ubiquitously expressed and serves as the primary intracellular superoxide dismutase. Mutations in SOD1, first linked to familial ALS in 1993 [48], cause disease through a toxic gain-of-function mechanism rather than loss of enzyme activity [49,50,51]. Wild-type SOD1 has been shown to incorporate into mutant aggregates [52] and even accelerates disease onset in some ALS models [53]. These findings imply that native SOD1 in the gut is unlikely to mitigate, and may even exacerbate, the toxic effects of hSOD1^G93A^.

The absence of pathology may also be attributed to the rapid turnover of gastrointestinal epithelial cells, which may serve as a specialized protective mechanism against protein aggregation. While neuron or muscle cells are long-lived, the epithelial layers of gut villi are completely replaced rapidly every 3–5 days in humans [54] and mice [55]. This creates a kinetic conflict for hSOD1^G93A^ pathogenesis: where SOD1 is a long-lived protein with a half-life of approximately 15.9 days in the central nervous system, its half-life in non-neuronal tissues is significantly shorter, such as 3.4 days in kidney or 1.5 days in liver [56]. Because the formation of toxic, insoluble aggregates is a time-dependent process [50,57], the rapid epithelial renewal likely functions as a ‘biological washout,’ shedding cells before mutant proteins can achieve the stable aggregation required to disrupt cellular homeostasis in gut villi. This distinguishes gut epithelium from the more static enteric nervous system, where a lack of turnover may allow for the gradual accumulation of toxic species over the lifespan of the organism.

There is substantial evidence linking gut dysbiosis and inflammation to the pathogenesis of ALS. While evaluating the gut microbiota and systemic cytokine milieu could offer deeper mechanistic insights, such changes are typically most informative when coupled with abnormalities in gut morphology, permeability, or neuromuscular function. Because our Gut-hG93A mice maintain normal intestinal morphology, permeability, body weight, lifespan and motor activity, it is unlikely that this model would harbor robust or causative shift in microbiota composition or inflammatory signaling at a baseline state. Any detectable changes (e.g., 16S rRNA sequencing) at this stage would likely be subtle and insufficient to drive measurable pathology. While our results demonstrate that gut epithelium-restricted hSOD1^G93A^ expression is insufficient to trigger a baseline ALS phenotype, they raise important questions regarding the ‘multi-hit’ hypothesis of neurodegeneration. Rather than a primary driver, the gut-restricted mutation may act as a priming factor. Future longitudinal studies utilizing 16S rRNA or metagenomic analysis are warranted, particularly in the context of environmental stressors—such as high-fat diets or inflammatory challenges (e.g., LPS administration)—to determine if a secondary ‘hit’ is required to catalyze the transition from a stable microbiome to a pathological, dysbiotic state.

Our findings suggest that hSOD1^G93A^ expression restricted to the gut epithelium is not primarily pathogenic. However, a key limitation of the Villin–Cre system is that it does not target other critical cell types, such as enteric neurons, immune populations, smooth muscle, or stromal compartments. Given that ALS-related gut dysfunction likely emerges from complex neuro-immune–microbiota interactions, the absence of a phenotype in our model may stem from the lack of mutant protein burden in these supportive and signaling compartments. Specifically, the enteric nervous system (ENS) and resident immune cells are known to play pivotal roles in maintaining gut homeostasis and modulating systemic inflammation. While our study successfully isolates the epithelial barrier, it cannot exclude the possibility that co-expression across multiple gut layers is required to replicate the pathology seen in systemic models. Therefore, this Gut-hG93A model should be viewed as a rational first step that narrows the search for primary pathogenic drivers, pointing toward the necessity of exploring non-epithelial compartments in future ALS gut research, such as using broader Cre-drivers (e.g., Wnt1-Cre for the enteric nervous system or Vav1-Cre for immune populations) to reconstruct the full gut–brain axis pathology.

In the present study, we prioritized functional neuromuscular endpoints—including motor performance, in vivo muscle contractility, and survival—which are the primary benchmarks for ALS-relevant disease progression. The lack of deficits across these measures in Gut-hG93A mice provides strong evidence that epithelial-restricted hSOD1^G93A^ expression does not lead to clinically significant pathology. However, we acknowledge that this study did not include histological analysis of the spinal cord or brain. Therefore, we cannot exclude the possibility of subtle, subclinical neuropathology, such as mild gliosis or the formation of small protein aggregates that did not reach a threshold for functional impairment. Future studies utilizing high-resolution immunohistochemistry or transcriptomic profiling of the spinal cord anterior horn will be valuable to determine if the gut-restricted mutation initiates latent molecular changes in the CNS that precede overt motor neuron loss.

Finally, this work aligns with ALS/MND (motor neuron disease) preclinical research guidelines [58], which emphasize the importance of publishing negative findings to clarify the true impact of candidate disease modifiers and mechanisms underlying ALS pathogenesis. The findings and their implications should be discussed in the broadest context possible. Future research directions may also be highlighted.

## 5. Conclusions

In this study, we demonstrated that expressing the ALS-associated hSOD1^G93A^ mutation exclusively within the intestinal epithelium at physiological levels is insufficient to disrupt tight junction integrity or intestinal permeability. These Gut-hG93A mice exhibited no significant deficits in lifespan, body weight, or comprehensive neuromuscular function. These findings suggest that the intestinal epithelium is not a primary, autonomous driver of ALS pathogenesis when mutant protein burden remains at physiological ratios. The lack of pathology may be attributed to the rapid turnover of intestinal epithelial cells, which likely prevents the stable accumulation of toxic aggregates: a process we term “epithelial proteostasis”. However, these results do not exclude the possibility that higher transgene dosages or the involvement of non-epithelial compartments—such as the enteric nervous system, smooth muscle, or resident immune populations—are required to initiate gut-driven neurodegeneration. Ultimately, this study provides a critical baseline for the “multi-hit” hypothesis of ALS. By establishing that gut epithelial hSOD1^G93A^ is not independently pathogenic, we identify this model as a latent genetic substrate. Rather than being discarded, this Gut-hG93A model serves as a valuable strategic tool for future investigations into the gut–brain axis, specifically for testing how environmental or secondary stressors may act as the necessary “second hit” to catalyze systemic disease.

## Data Availability

All the data are available in the article.

## Supplementary Materials

The following supporting information can be downloaded at: https://www.mdpi.com/article/10.3390/biom16020253/s1, Figure S1: Original images for Figure 1B–D; Figure S2: Original images for Figure 3A,C.

## Abbreviations

The following abbreviations are used in this manuscript:

ALS	Amyotrophic Lateral Sclerosis
CNS	Central Nervous System
SOD1	Cu/Zn superoxide dismutase type 1
RFID	Radio-frequency-identification
FD4	Fluorescein isothiocyanate (FITC)-4KD
R70	Rhodamine B isothiocyanate
TA	Tibialis Anterior
