# Supplementary material for: Absence of Neuromuscular Dysfunction in Mice with Gut Epithelium-Restricted Expression of ALS Mutation hSOD1G93A
Source: Biomolecules. 2026 Feb 5;16(2):253. doi: 10.3390/biom16020253 (PMC12938467; doi:10.3390/biom16020253)

Figure S1: Original images for Figure 1B-D

Original Western blot for Figure 1B

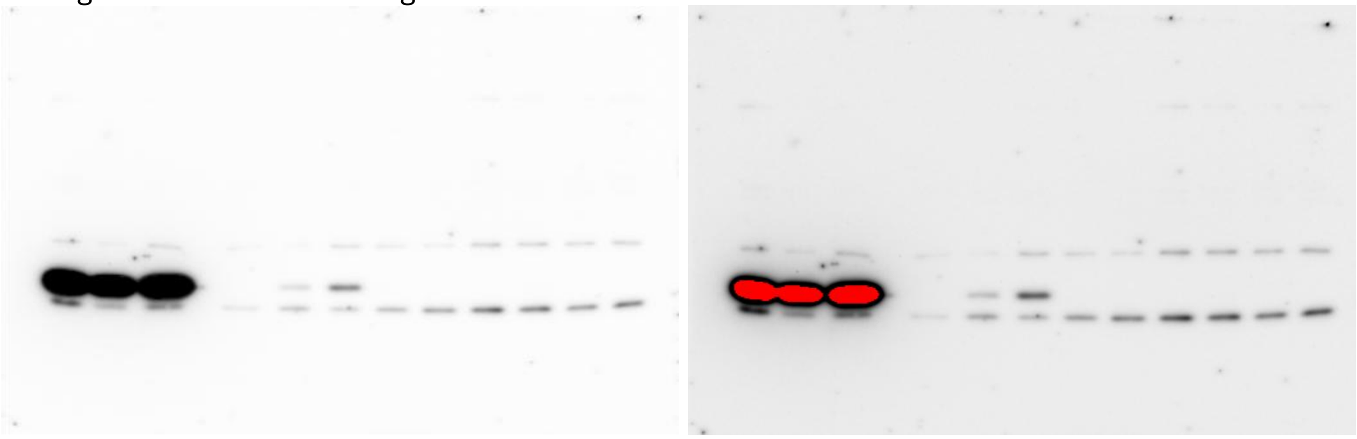

Original Western blot for Figure 1C

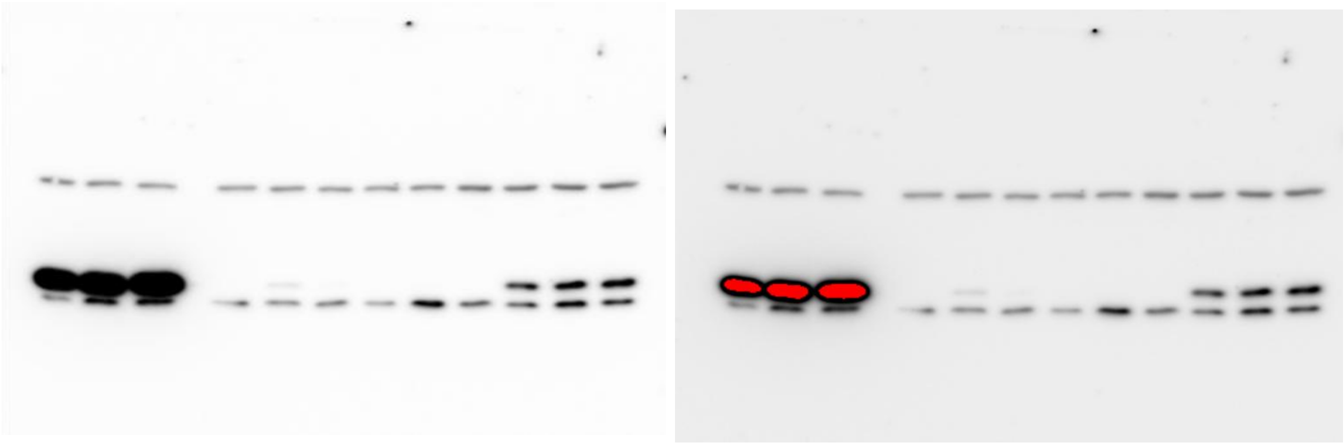

Original image for Figure 1D

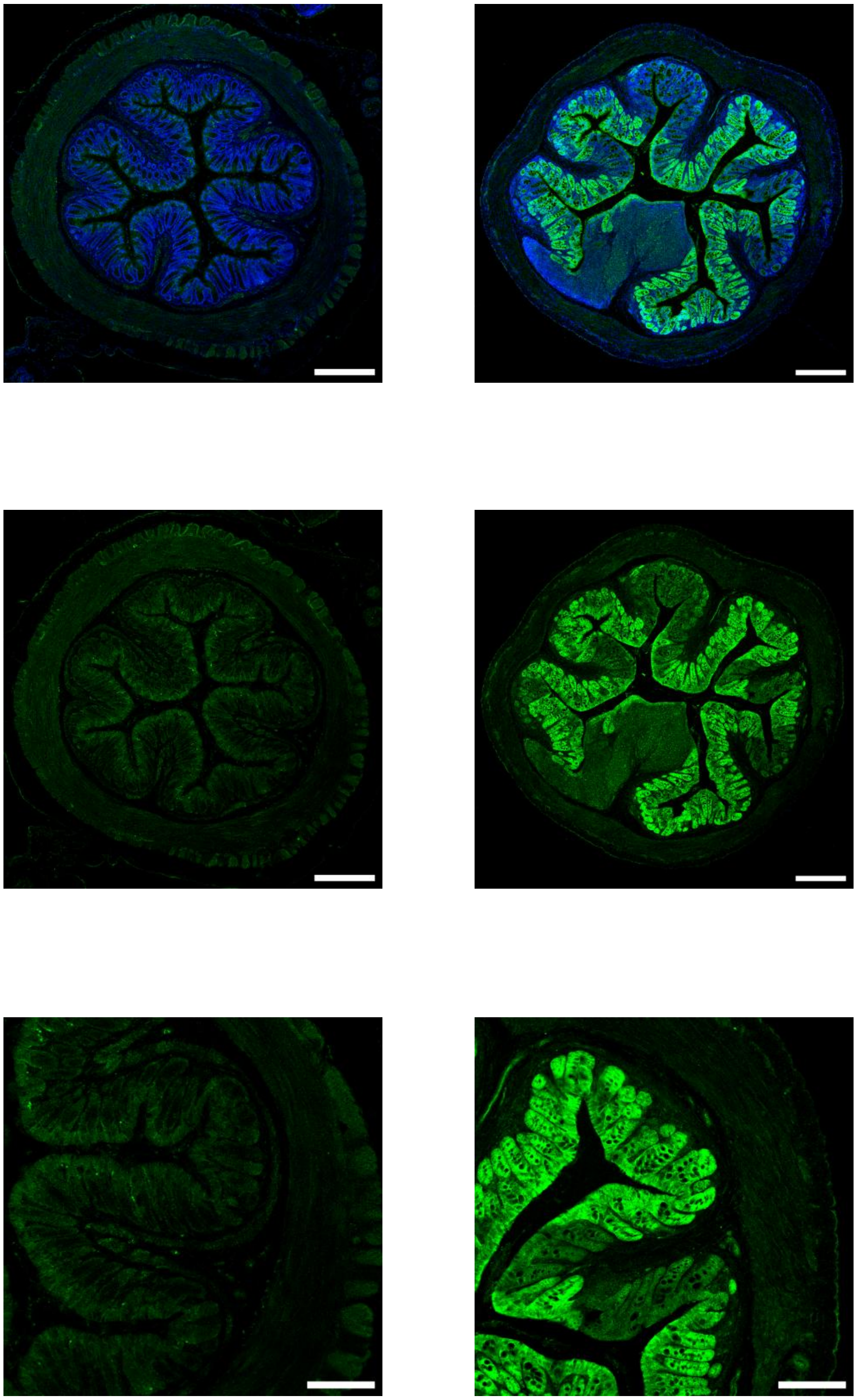

Figure S2: Original images for Figure 3A,C

Original western blot for Figure 3A

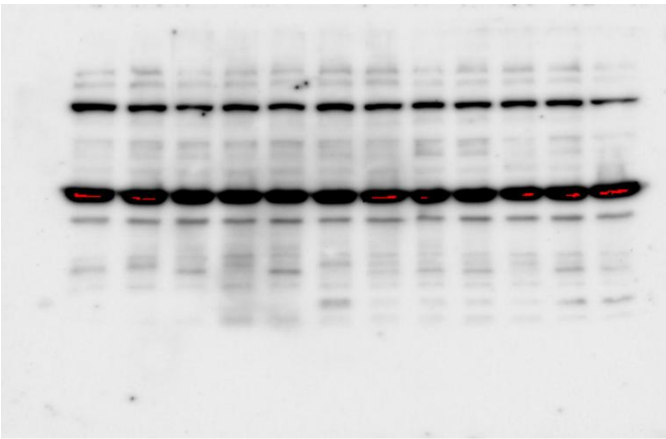

ZO-1

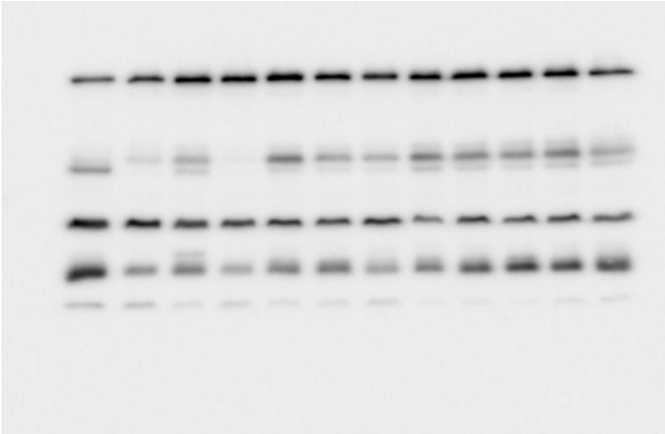

E-cadherin

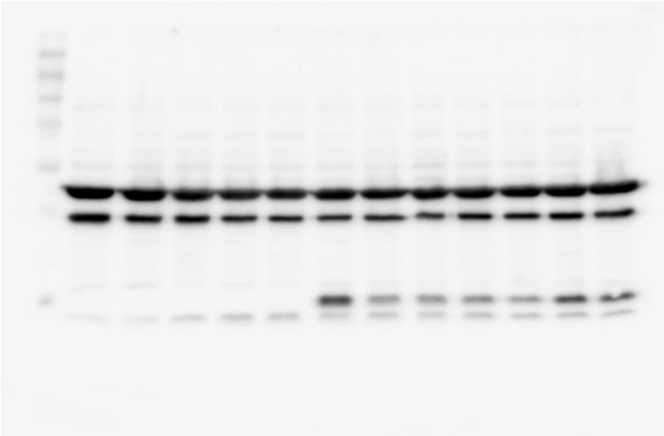

GAPDH

Original images for Figure 3C

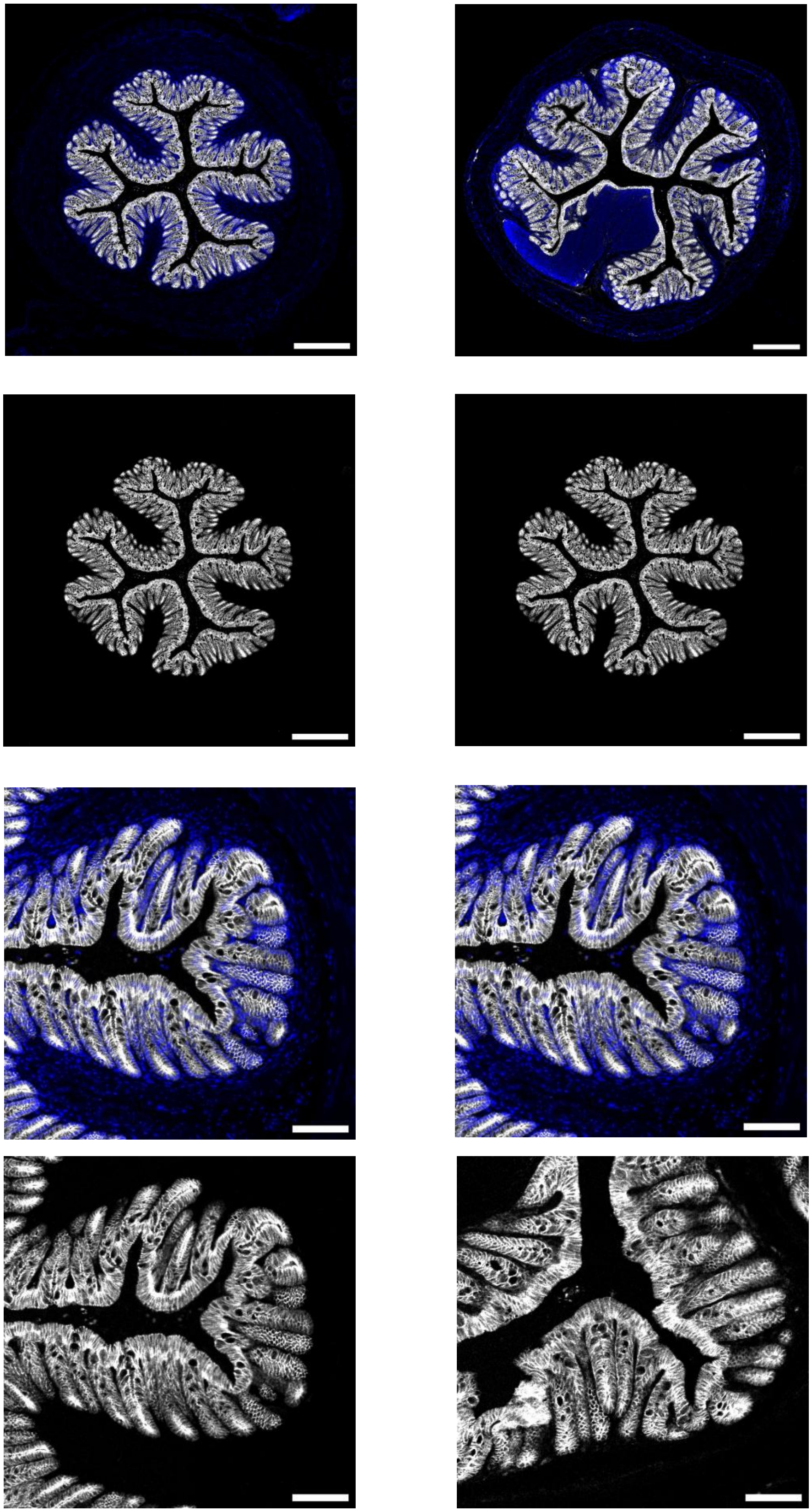

Supplement: Supplementary file 1 [file biomolecules-16-00253-s001.zip › biomolecules-4080924-supplementary.pdf]
